# Anaphylaxis to supplemental oral lactase enzyme

**DOI:** 10.1186/s13223-016-0171-8

**Published:** 2016-12-13

**Authors:** M. R. Voisin, R. Borici-Mazi

**Affiliations:** 1 School of Medicine, Faculty of Health Sciences, Queen’s University, Kingston, ON K7L 3N6 Canada; 2 Division of Allergy and Immunology, Department of Medicine, Queen’s University, 166 Brock Street, Kingston, ON K7L 5G2 Canada

**Keywords:** Anaphylaxis, Lactase allergy, *Aspergillus*, Lactose intolerance, IgE mediated

## Abstract

**Background:**

Anaphylactic reactions involving IgE mediated hypersensitivity have been frequently reported for a number of uncommon foods. However, cases of anaphylaxis to over the counter vitamins and oral supplements have been rarely published. Lactose intolerance affects approximately 20% of Canadians and roughly 70% of the world’s population of any age. Lactose intolerance develops primarily due to the absence of the enzyme lactase and treatment involves avoidance of lactose-containing foods or ingestion of commercially available lactase enzyme preparations prior to their consumption. This case report represents the first documented evidence of anaphylaxis after exposure to supplemental lactase enzyme preparation.

**Case presentation:**

A 38 years old Caucasian female presented with a history of self-diagnosed adult-onset lactose intolerance and a suspected allergy to lactase containing tablets. She reported an episode of bilateral orbital swelling, shortness of breath, and throat constriction after oral ingestion of a supplemental lactase enzyme tablet. Her symptoms slowly resolved with the administration of inhaled salbutamol and oral diphenhydramine. She handled lactase tablets for years to her children who were lactose intolerant, but had never ingested the tablets herself prior to the reported episode. In clinic, physical examination was benign, and skin prick testing to a slurry of the lactase tablet revealed a strongly positive reaction wheal size of 10 mm and flare of 60 mm with normal controls. The patient reported throat tightness and constriction after skin prick testing and required cetirizine treatment and observation in clinic. Subsequent skin testing was performed with individual ingredients of the lactase tablet provided by the manufacturer and *Aspergillus niger*, a common bacteria used in lactase preparations. Only concentrated lactase enzyme elicited a positive response. The patient was diagnosed with lactase tablet induced anaphylaxis due to synthetic lactase enzyme IgE mediated allergy, and was advised to avoid all products containing lactase enzymes as an ingredient and to carry an epinephrine auto-injector.

**Conclusion:**

This is the first documented case report of an anaphylactic reaction to supplemental lactase enzyme. This case report reinforces the importance of thorough allergy assessment, education on avoidance of triggers, in particular with uncommon allergens.

## Background

Anaphylactic reactions involving IgE mediated hypersensitivity have been frequently reported for a number of uncommon foods; however, cases of anaphylaxis to over the counter oral supplements and vitamins have been rarely published. Lactose intolerance results from lack of the enzyme lactase, leading to bloating, flatulence and diarrhea after the ingestion of lactose-containing foods. The prevalence of lactose intolerance varies considerably depending upon ethnic background, with overall rates approaching 20% [1]. Lactose intolerance can develop in two main patterns; primary or secondary, with congenital lactase deficiency being a third extremely rare cause [2, 3]. Management of lactose intolerance focuses primarily on avoidance of lactose-containing foods or ingestion of commercially prepared lactase enzyme preparations, often produced by subspecies of *Aspergillus* [4]. Although previous reports of allergic reactions to supplemental lactase either via ingestion or occupational exposure have been published, this is the first case report of a systemic, anaphylactic reaction in a patient after first ingestion of a supplemental lactase enzyme preparation [5–9].

## Case presentation

A previously healthy 38 years old Caucasian female presented to the outpatient allergy clinic in the fall of 2014 with self-diagnosed adult-onset lactose intolerance and a suspected allergy to a commercial available lactase enzyme tablets. She reported sudden onset of bilateral orbital swelling and facial urticaria, and rapidly worsening throat constriction and shortness of breath after her first oral ingestion of a supplemental lactase tablet. She took two tablets of diphenhydramine and 4 puffs of a salbutamol puffer which relieved her throat constriction and shortness of breath within 20–30 min. The bilateral orbital swelling slowly decreased within next 24 h with additional doses of oral diphenhydramine. She had avoided ingestion or handling of the supplemental lactase tablets since this reaction. Upon careful review of the history, patient had experienced recurrent itchiness and urticarial lesions in her hands and face after having been in contact with the supplemental lactase tablets she handed to her kids. She had no history of prior ingestion of any commercial supplemental lactase-containing foods.

Patient’s past medical history included well-controlled mild intermittent asthma, oral allergy syndrome to raw fruits and vegetables, allergic rhinitis, GERD, and hypertension. She reported a self-diagnosis of lactose intolerance approximately twelve months prior to her reported episode of anaphylaxis. Her medication list included ranitidine, fluticasone nasal spray, and salbutamol as needed. She reported that her children were diagnosed with lactose intolerance and she was handling supplemental lactase tablets for years with no reactions, prior to the initiation of her skin symptoms a few months earlier.

**Fig. 1:**
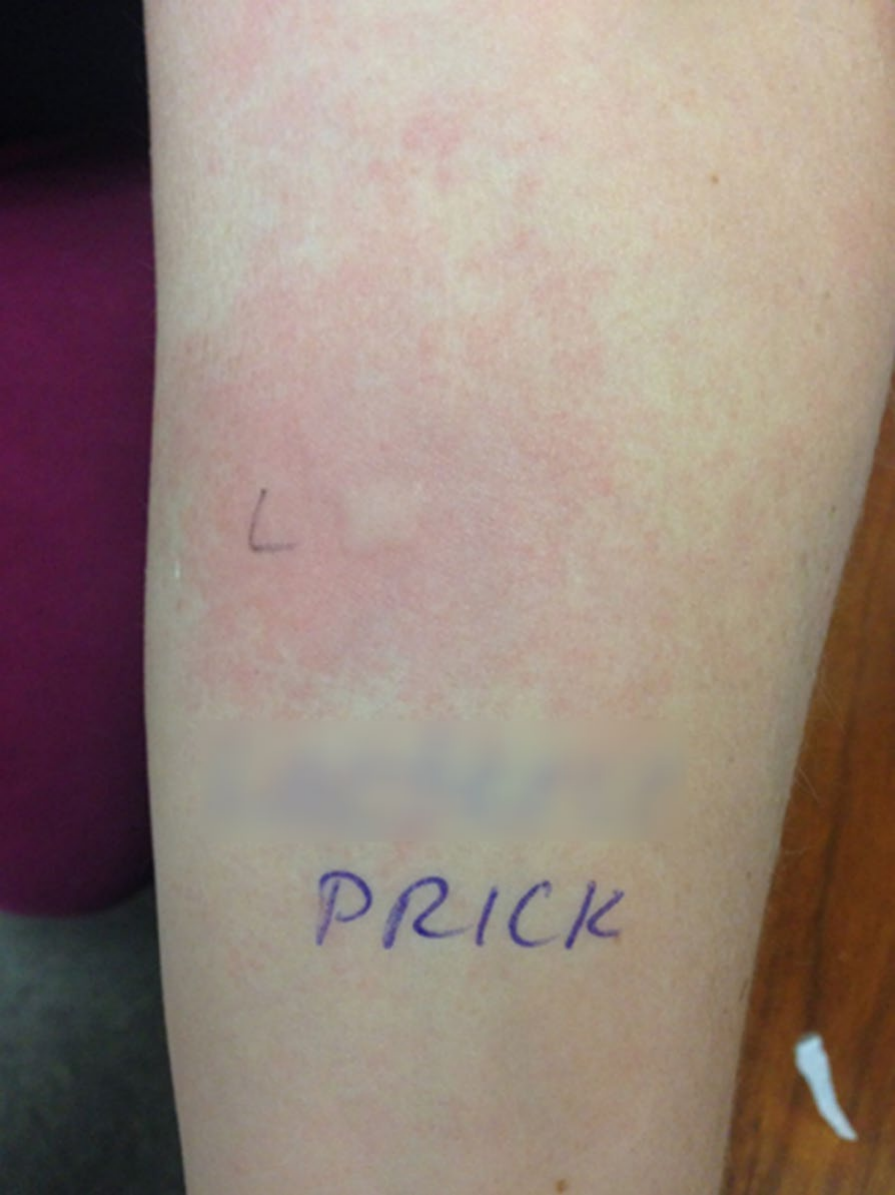
Skin test response to a slurry of lactase enzyme tablet applied to patient’s forearm

Upon assessment in clinic, physical examination was unremarkable. Skin prick testing (SPT) to environmental and food allergens was performed using allergens from Omega Laboratories, Montreal Quebec. SPT was positive for dust mites, trees, grasses, ragweed, and molds of *Alternaria and Cladosporium,* but it was negative for cow’s milk, egg, wheat, soy, sesame, inhalant *Aspergillus species* and subspecies of *Aspergillus niger*. SPT to subspecies of *Aspergillus oryzae* was not performed due to lack of allergenic extract. SPT to a saline slurry of the crushed lactase tablet (approximately 10% weight/volume) revealed a positive wheal and flare reaction, longest diameters of wheal and flare were 10 and 60 mm, respectively, with adequate controls (Fig. 1). After skin testing was completed, patient reported sudden onset of throat tightness which resolved after treatment with oral cetirizine 20 mg and 2 puffs of inhaled salbutamol. During a subsequent office visit, the patient underwent skin testing to ingredients of the lactase tablet provided by the manufacturer. Skin testing was strongly positive for a slurry of approximately 10% weight/volume of the lactase enzyme (4000 FCC lactase units) and negative for the other non-enzyme ingredients of the tablet including dextrose, microcrystalline cellulose, calcium stearate and natural mint flavour. Skin testing to the crushed lactase tablet and its ingredients was performed in similar fashion in two healthy volunteers and it was entirely negative with appropriate controls.

Patient was diagnosed with anaphylaxis caused by supplemental lactase tablet due to synthetic lactase enzyme IgE mediated allergy. She was advised to avoid handling and ingestion of all products containing lactase enzymes as an ingredient and to carry an epinephrine auto-injector. She has had no further accidental exposures. Subsequently, patient was gradually introduced to regular dairy products and has remained asymptomatic, without the need for further investigations.

## Discussion

This report describes the case of a 38 years old female who reported systemic anaphylactic symptoms after her first oral ingestion of supplemental lactase enzyme-containing tablet. Enzymes are known to be high molecular weight sensitizers, and evidence of allergic symptoms has been previously reported most commonly as respiratory allergic symptoms including asthma and/or rhinitis at variable levels of exposure [7]. Previous studies have described occupational rhino conjunctivitis, asthma, and contact dermatitis in pharmaceutical workers exposed to lactase powder for commercial use [4, 8, 9]. The earliest study reported lactase enzyme sensitization in 31% or 65 of 207 workers involved in the handling of lactase containing products [9]. Another study elaborated on a cross-sectional survey of 94 pharmaceutical workers exposed to lactase, of which 29% or 27 had positive skin testing [4]. Finally, a study involving 13 employees at a lactase tablet manufacturing plant demonstrated lactase sensitization in 69% or 9 employees [8]. Unlike these reports, our patient developed solitary skin involvement, but denied respiratory symptoms of rhino conjunctivitis or asthma after repeated dermal exposures that occurred during handling of lactase enzyme tablet. Instead, she experienced involvement of respiratory tract as part of systemic anaphylactic reaction after her first oral ingestion of supplemental lactase enzyme.

In addition to studies describing occupational exposure to lactase enzyme preparations, there have been two case reports in the literature documenting more specific allergic reactions to lactase enzyme [5, 6]. The earliest case report by Binkley [5], described an IgE mediated allergic reaction to *Aspergillus oryzae* derived lactase, with the patient experiencing allergic symptoms confined to the oropharynx after oral ingestion of lactase supplements. The author suggested that the sensitization of this patient occurred either due to prior exposure to cross-reacting *Saccharomyces fragilis* found in the specific brand of lactase tablet, or as a result of the patient’s inhalant allergy to *Aspergillus* species, with a similar mechanism to an “oral allergy syndrome”. Unlike this case report, our patient did not demonstrate an IgE mediated sensitivity to inhalant *Aspergillus species* and she also denied known previous consumption of other supplemental lactase products to account for the sensitization. Therefore, to the best of our knowledge, the most likely route of sensitization to synthetic lactase enzyme was with repeated prior dermal exposure when handling lactase tablets to her children. Eventually she experienced contact urticaria when handling the lactase tablets and later on suffered a systemic reaction compatible with anaphylaxis upon first oral ingestion of the lactase tablet. The sensitization pattern of our patient was similar to the case report published in 2007 by Laukkanen et al. [6]. He described the presence of serum lactase specific IgE antibodies in a pharmaceutical worker exposed to powdered form of lactase enzyme who suffered from contact dermatitis and allergic rhino conjunctivitis. However, in our case report, confirmation of IgE mediated sensitization was obtained via positive SPT to the slurry of crushed lactase tablet and concentrated lactase enzyme, as well as lack of sensitivity to other non-lactase ingredients of the lactase tablet. The negative SPT responses in two healthy volunteers and the appropriate controls we obtained during the SPT procedure confirmed that positive skin tests were specifically attributed to IgE mediated sensitivity to lactase enzyme and not due to skin irritation. In addition, although to a milder degree, patient experienced similar symptoms in clinic after the positive skin testing with the crushed lactase tablet confirming that lactase enzyme allergy was the culprit.

Pharmaceutical molds are widely used in industry as a source for producing supplemental lactase enzyme preparation. In our case report, the manufacturer confirmed that the lactase enzyme concentrate used to make the lactase tablet was derived from cultures of *Aspergillus oryzae*. *Aspergillus oryzae* is an aerobic, filamentous fungus with many applications in the food industry including its traditional use in China and Japan to produce koji, a complex enzyme preparation used in the production of soy sauce, miso, and sake [10, 11]. Its ability to secrete large amounts of proteins has facilitated its use in modern biotechnology including large scale production of enzymes including lactase [12]. Antigenic determinants identified from Aspergillus include α-amylase—the major cause of Baker’s asthma—and lipase, and previous reports of sensitization were thought to be due to repeated inhalational exposure to the enzyme [4, 5, 11]. Since our patient did not demonstrate IgE mediated sensitivity to inhalant *Aspergillus* species upon SPT, but reacted to the lactase ingredient derived from *Aspergillus oryzae*, the likely mechanism of sensitization remains repeated dermal exposure to the same lactase tablet, but possible prior oral consumption of foods that may have contained lactase enzyme derived from *Aspergillus oryzae* cannot be excluded. The patient was educated about the risk of future accidental exposures with consumption of supplemental lactase-containing products and the need to carry an epinephrine auto injector, although she denied any allergic-like symptoms outside of the context of the above mentioned episodes.

Finally, this patient was self-diagnosed with lactose intolerance. Subsequently to our assessment, she was able to re-introduce the dairy products in her diet. Therefore, there was no need for further investigation of lactose intolerance and future consumption of alternate supplemental lactase enzyme.

## Conclusion

This case represents the first report of a systemic anaphylactic reaction in a patient with self-diagnosed lactose intolerance after oral ingestion of a supplemental lactase tablet, resulting from an IgE mediated allergy to lactase enzyme. This study highlights the potential for serious and potentially life-threatening anaphylactic reactions to commonly used over the counter supplements and a need for patients and healthcare providers to be aware of rare but significant allergies to a variety of uncommon allergens. Patients presenting with a history of allergic and/or anaphylactic symptoms should be screened by both a thorough history and adequate allergy testing and be educated about the risks of future exposures and treatment of potential anaphylactic reactions.
